# Comparison of the Initiation Time of Enteral Nutrition for Critically Ill Patients: At Admission vs. 24 to 48 Hours after Admission

**DOI:** 10.1155/2021/3047732

**Published:** 2021-09-17

**Authors:** Anshan Yu, Yanmei Xie, Meixin Zhong, Fen Wang, Huachun Huang, Liang Nie, Xiaofeng Liu, Mingfang Xiao, Hongquan Zhu

**Affiliations:** Department of Critical Care Medicine, The First Affiliated Hospital of Gannan Medical University, Ganzhou, Jiangxi 341000, China

## Abstract

**Objective:**

To investigate the better time of initiation of enteral nutrition for critically ill patients, such as at admission or 24 to 48 hours after admission.

**Methods:**

This was a prospective, randomized, parallel-controlled, single-blind, interventional clinical trial. A total of 100 patients admitted to the intensive care unit (ICU) of our hospital between January 2017 and December 2018 were recruited in this study. These patients had been divided into the control group or intervention group by a computer-generated random number table, and each group had 50 patients. For the control group, a gastric tube was inserted to start enteral nutrition at 24 to 48 hours after admission. For the intervention group, a nasojejunal tube was placed to start enteral nutrition at admission. The main endpoints included serum albumin and prealbumin at admission and on days 3, 7, and 14 after admission, length of ICU stay, ventilator time, and complications such as diarrhea, gastric retention, esophageal reflux, and pulmonary infection.

**Results:**

The results showed that serum albumin and prealbumin were significantly higher in the intervention group than in the control group (*P* < 0.05). The length of ICU stay (*P* < 0.05) and ventilator time (*P* < 0.05) were both significantly shorter in the intervention group than in the control group. The incidences of gastric retention, esophageal reflux, and pulmonary infection were significantly lower in the intervention group than those in the control group (*P* < 0.05).

**Conclusion:**

In the absence of contraindications, enteral nutrition can be initiated immediately after admission to the ICU (within 6 hours), and feeding nasojejunal tube is recommended. It can improve the nutritional status and prognosis of critical patients, improve the feeding effect, shorten the length of stay in the ICU and the use of the ventilator, and reduce the incidence of complications.

## 1. Introduction

Insufficient nutrition is very common in critically ill patients admitted to the intensive care unit (ICU), and it is important to strengthen nutritional support for these patients. Enteral nutrition is the first choice of nutritional support in critically ill patients [1, 2]. For these patients, early enteral nutrition is critical for restoring normal intestinal permeability, preventing intestinal infection and intestinal failure, and improving outcomes [3, 4]. The initiation time and feeding route of enteral nutrition are the focus of current research. Regarding the time of initiation of enteral nutrition, most current guidelines and studies recommend that enteral nutrition be initiated at 24 hours after admission [5–7]. However, studies have shown that this regimen still leads to feeding delays.

In a multicenter study, the average enteral nutrition initiation time in ICU patients was 46.5 hours [8]. Delayed feeding leads to insufficient intake and malnutrition in patients, which further leads to poor prognosis and increased mortality [9, 10]. Several previous studies have investigated the benefits of earlier enteral nutrition [11–13]; in these studies, enteral nutrition was initiated as early as 4 hours after admission. However, the studies were conducted in patients with obstructive jaundice and burns, respectively, and did not include other critically ill patients. Therefore, the study calls for improving patient feeding to be the focus of quality care in the ICU [14].

This study was designed to investigate the initiation of enteral nutrition at admission versus at 24 to 48 hours after admission in critically ill adult patients, so as to find the best enteral nutrition initiation time and nutrition pathway for patients with severe illness, reduce feeding delay, and improve nutrition and prognosis of patients with severe illness.

## 2. Materials and Methods

### 2.1. Study Design

This was a prospective, randomized, parallel-controlled, single-blind, interventional clinical trial.

### 2.2. Subjects

From January 2017 to December 2018, patients admitted to the intensive care unit (ICU) of our hospital were enrolled in this study. These patients had been divided into the control group or intervention group by a computer-generated random number table. For the control group, a gastric tube was inserted to start enteral nutrition at 24 to 48 hours after admission. For the intervention group, a nasojejunal tube was placed to start enteral nutrition at admission. This study was conducted in accordance with the Declaration of Helsinki and approved by the ethics committee of our hospital. All participants had signed informed consent.

### 2.3. Inclusion and Exclusion Criteria and Group Assignment

The inclusion criteria were as follows: (1) patients admitted to the general adult ICU; (2) patients who were ≥14 years of age; (3) patients who have signed informed consent.

The exclusion criteria were as follows [5]: (1) hemodynamic instability (mean arterial pressure <60 mm Hg); (2) contraindications for enteral nutrition due to functional, structural, or physiological gastrointestinal disorders such as intestinal ischemia or obstruction, gastrointestinal perforation or fistula, abdominal compartment syndrome, or active upper gastrointestinal bleeding; (3) other contraindications for enteral nutrition, such as selective enteroscopy or bowel surgery.

### 2.4. Calculation of Sample Size

Sample size was calculated as *G*^*∗*^power (two independent sample *t-*tests, two-sided, significance level of 0.05, statistical power of 0.8, and effect size of 0.6217 calculated with means and deviations from the two groups in the pilot study). The result showed that 84 subjects were required, and 100 subjects should be enrolled considering a 20% exclusion rate.

### 2.5. Group Assignment

A computer-generated random number table was used for randomization. The patients were assigned an ID number from 1 to 100 based on the time of admission, and SPSS v 25.0 was used to generate a random number associated with each patient ID. Patients with a random number from 1 to 50 were assigned to the intervention group, and those with a number from 51 to 100 were assigned to the control group, resulting in 50 patients in each group. The randomization process was performed by a designated member of the study team. The assignment results were sealed in an opaque envelope and were not disclosed to the data collection staff.

### 2.6. Interventions

#### 2.6.1. Tube Materials

A silicone enteral nutrition tube with a guide wire (F12) was used in both groups. The tube length was 80 cm in the control group and 145 cm in the intervention group, with 20 cm of extra length.

#### 2.6.2. Training

A tube-insertion nurse team and an observation nurse team were established and trained. The two teams were independent of each other and had different members. The first team inserted the tube; and the second team, which was blinded to group assignment, recorded all outcome measures.

#### 2.6.3. Tube Insertion and Management

In the control group, the tube was inserted into the stomach, with a length of about 60 cm from the nose to the stomach 24 to 48 hours after admission (insertion length: 60 cm, extra: 20 cm; both segments were labeled “enteral nutrition tube”). For the intervention group, a tube was inserted into the duodenum with a length of 125 cm from the nose to the duodenum (no more than 6 hours after admission) (insertion length: 125 cm (confirmed with ultrasound to have passed the pylorus) [15], extra: 20 cm; both segments were labeled “enteral nutrition tube”). Both groups received routine enteral nutrition management. After insertion, the observation nurses provided management and recorded all outcome measures. For patients with no contraindications, the head of the bed was raised by 30°. Daily enteral nutrition (Nutrison Fibre, Nutricia, Netherlands, 1 kcal/mL) was started at 20 mL/h (20 kcal/h) and infused at a constant rate with a nutrition pump. The gastric contents were aspired every 4 hours. If the volume of the aspired contents was less than 150 mL or less than the volume of infusion over 4 hours, the infusion rate was increased by 20 mL/h until the feeding target (30 kcal/kg/d) was reached in 2 days. If the aspired contents were no less than 150 mL or the volume of infusion over 4 hours (indicating gastric retention) or if diarrhea and vomiting occurred, the infusion rate was reduced by 50%. After 4 hours, if the volume of the aspired gastric content was still no less than the volume infused over 4 hours or if vomiting persisted, feeding was terminated.

### 2.7. Outcome Measures

The primary outcome measures included serum albumin and prealbumin at admission and on days 3, 7, and 14 after admission. The secondary outcome measures included the length of ICU stay, ventilator time, and complications during the first 14 days in the ICU.

The complications included diarrhea, reflux aspiration, gastric retention, and pulmonary infection. The complications were evaluated as follows [16]: (1) diarrhea: if the frequency of bowel movements was equal to or greater than 4 per day or was increased by 2 times per day, diarrhea was considered; (2) gastric retention: gastric retention was considered if the volume of the aspired gastric contents (every 4 hours) was no less than 150 mL or no less than the volume infused over 4 hours; (3) pulmonary infection: pulmonary infection was considered if there were new signs of inflammation on the chest X-ray with any two of the following symptoms: cough, breathing difficulty, fever, leukocytosis, wheezing, and rales per auscultation.

### 2.8. Statistical Analysis

We used the software program SPSS 25.0 (IBM, Chicago, USA) to conduct the statistical analysis. The continuous variables of the normal distribution were expressed as mean ± standard deviation, the continuous variables of the nonnormal distribution were expressed as median (interquartile range (IQR)), and the categorical variables were expressed as frequency (percentage (%)). The Kolmogorov–Smirnov (KS) test was used to analyze the normality of continuous data. Normally distributed data were analyzed with the two independent sample *t-*tests; nonnormally distributed data were analyzed with Wilcoxon's rank-sum test. Categorical data were analyzed with the *χ*^*2*^ test. For repeated measurements, normally distributed data were analyzed with the analysis of variance of repeated measurements and the general linear model (processing effect, time effect, and the interaction between the two); nonnormally distributed data were analyzed with generalized estimating equations. *P* < 0.05 was considered statistically significant.

## 3. Results

### 3.1. Drop-Out and Exclusion Criteria

A total of 100 patients had been recruited. However, a total of 5 patients were withdrawn from the study due to less than 72 hours of hospital stay, including 3 in the control group and 2 in the intervention group. A total of 8 patients were withdrawn from the study due to intravenous albumin infusion during the observation period, including 4 in the control group and 4 in the intervention group. Finally, 44 patients in the intervention group and 43 in the control group (*N* = 87) completed the study.

### 3.2. The General Characteristics

A total of 87 patients who were admitted to the ICU of our hospital between January 2017 and December 2018 were enrolled in this study, including 44 patients in the intervention group and 43 patients in the control group. There were 59 males and 28 females, aged 15 to 88 years (mean: 56.8 ± 16.8). No significant between-group differences were observed in disease composition and age, sex (Table 1), or baseline data such as APACHE II score at admission, albumin at admission, and prealbumin level at admission (Table 2).

In consideration of the influence of etiology on the outcome, we analyzed the etiology of the two groups of patients, and the results showed (see Table 1) that there was no difference in etiology between the control group and the intervention group. In order to analyze the effects of different disease severities and different intestinal functions on the outcome indicators, we compared the two groups, and the results showed (see Table 2) that there were no differences between the control group and the intervention group in disease severity, preintervention albumin, and prealbumin.

### 3.3. Changes in Mean Albumin over Time between Two Groups

**Figure 1:**
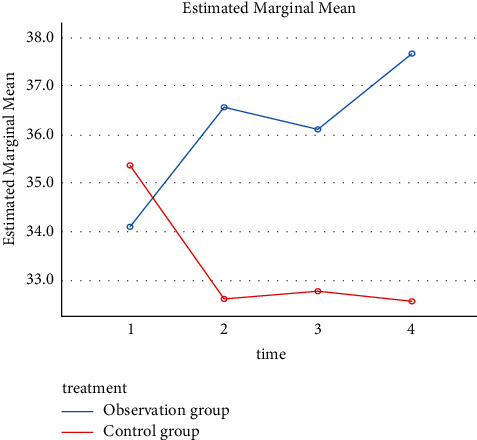
Line chart of the changes in mean albumin over time.

The results showed that the albumin level was initially similar between the two groups. Over time, the value increased in the intervention group, while it decreased and then remained low in the control group. Mauchly's sphericity test showed that, for the interaction item treatment ^*∗*^ time, the dependent variable did not comply with the sphericity hypothesis (*χ*^2^ = 22.379, *P* ≤ 0.001, and Greenhouse–Geisser epsilon = 0.508) and thus required Greenhouse–Geisser correction. The details are shown in Figure 1.

The results showed that the corrected interaction effect of treatment ^*∗*^ time was *F* (1.524, 22.855) = 3.033 (*P* = 0.08), indicating no statistical significance or the absence of an interaction, and the main effect of treatment on albumin was statistically significant (*P* = 0.039) (Table 3). The albumin level was higher in the intervention group than in the control group by 2.761 g/L (95% confidence interval (CI): 0.157–5.365). There was no significant effect on albumin (*P* = 0.809).

### 3.4. Changes in Prealbumin Levels between Two Groups

Based on the intercept (0.187, *P* < 0.001) parameter estimate and test, the mean initial prealbumin level was significantly higher than zero in the two groups. In the intervention group, the prealbumin level changed at a rate of 0.002 over time, indicating a slight but insignificant increase in the prealbumin level (*P* = 0.148). The prealbumin level was higher in the intervention group than in the control group by 0.048 mg/L (*P* = 0.002). No significant interaction effect was observed between the treatment time and group (*P* = 0.598). The details are listed in Table 4.

### 3.5. Length of ICU Stay and Ventilator Time between Two Groups

In the intervention group, the length of ICU stay was 6.4 (3.3, 10.7) days, and the ventilator time was 3.6 (1.2, 6.0) days. In the control group, the length of ICU stay was 7.9 (5.6, 12.1) days, and the ventilator time was 5.5 (4.1, 8.8) days. The results showed that the length of ICU stay and ventilator time were significantly shorter in the intervention group than in the control group (*P* < 0.05). The details are listed in Table 5.

### 3.6. Complications between Two Groups

In the control group, 5 cases (11.6%) had diarrhea, 5 cases (11.6%) had gastric retention, and 7 cases (16.3%) had pulmonary infection. In the intervention group, 2 cases (4.5%) had diarrhea, no case (0.0%) had gastric retention, and 1 case (2.3%) had pulmonary infection. The results showed that no significant between-group difference was observed in the incidence of diarrhea. The incidences of gastric retention and pulmonary infection were significantly lower in the intervention group than in the control group (*P* < 0.05). The details are listed in Table 6.

## 4. Discussion

Enteral nutrition refers to the provision of nutrients through the gastrointestinal tract to maintain the body's metabolism. The gastrointestinal tract plays an important role in immune defense. Compared with parenteral nutrition, enteral nutrition not only has the advantages of more physiologic absorption and utilization of nutrients but also helps maintain the integrity of the intestinal mucosal structure and barrier function.

Initiation delay is one of the causes of insufficient enteral nutritional intake in critically ill patients. The “Consensus of Early Enteral Nutrition Clinical Practice in Critically Ill Patients” [5] of 2018 states that enteral nutrition support should be provided as soon as possible after related contraindications are excluded. Clinical studies have shown that initiation delay is one reason why patients fail to reach the feeding target and that it has adverse effects on recovery [14, 17, 18]. One of the critical factors for ensuring that patients benefit from nutritional therapy is to reduce initiation delay in order to achieve the feeding target as soon as possible. Because of clinical practice and concerns about the poor tolerability of enteral nutrition by critically ill patients, clinicians often wait 24 hours after admission to decide whether to initiate enteral nutrition, which is the cause of initiation delay. Early initiation of enteral nutrition at admission reduces the observational wait time after admission, thereby reducing initiation delay and improving outcomes. This study showed that the initiation of enteral nutrition at admission improved serum albumin and prealbumin and reduced the length of ICU stay and ventilator time relative to the initiation of enteral nutrition at 24 to 48 hours after admission.

Due to technical and equipment limitations, via a nasogastric tube is still the most common method for providing early enteral nutritional support; however, sedative, analgesic, and vasoactive drugs are often used in critically ill patients, resulting in varying degrees of decreased gastric motility. Consequently, nasogastric feeding is associated with a high risk of aspiration and pulmonary infection. Its safety profile is less than satisfactory [19, 20]. Several studies [16, 21–23] have shown that nasogastric feeding is associated with a high incidence of feeding intolerance, which leads to high morbidity and mortality and prolonged ICU stays, with adverse effects on patient recovery. Nasojejunal feeding reduces aspiration and pneumonia, improves outcomes and gastrointestinal tolerability, and reduces complications and the time to reach the feeding target [24, 25], which allows enteral nutrition to be initiated early and safely. Early nutritional intervention by nasojejunal feeding can reduce the incidence of complications.

Fuentes Padilla et al. [26] reported that it was uncertain whether early enteral nutrition, compared with delayed enteral nutrition, affects the risk of mortality within 30 days, feed intolerance or gastrointestinal complications, or pneumonia due to very low-quality evidence. Tian et al. [27] reported that early enteral nutrition reduced mortality and pneumonia compared with delayed enteral intake, but there were no clear clinical advantages of early enteral nutrition over parenteral nutrition. However, our study found that early enteral nutrition can improve the nutritional status and prognosis of critical patients, improve the feeding effect, shorten the length of stay in the ICU and the use of the ventilator, and reduce the incidence of complications. In summary, for critically ill patients who require early enteral nutrition support and have no contraindications, enteral nutrition should be initiated immediately at admission (within 6 hours). Early nutritional intervention by nasojejunal feeding is advantageous for improving nutrition status, reducing the lengths of ICU stays and ventilator time, and promoting recovery.

This study still had several limitations: firstly, this was a small (*N* = 87), single-center study. Large multicenter studies are needed to further validate the results. Secondly, in this study, the measures were observed only through day 14 after admission, and there was no observation of long-term effects. Studies with a longer observation period are needed to further validate the results.

## 5. Conclusion

In the absence of contraindications, enteral nutrition can be initiated immediately after admission to the ICU (within 6 hours), and feeding nasojejunal tube is recommended. It can improve the nutritional status and prognosis of critical patients, improve the feeding effect, shorten the length of stay in the ICU and the use of the ventilator, and reduce the incidence of complications.

## Figures and Tables

**Table 1:** Disease composition (*n*, %).

Diagnosis	Cerebral infarction with respiratory failure	AECOPD	Postbrain surgery	Multitrauma	Others	Total
Observation group	15 (34.1)	7 (15.9)	8 (18.2)	7 (15.9)	7 (15.9)	44 (100)
Control group	8 (18.6)	14 (32.6)	9 (20.9)	3 (7.0)	9 (20.9)	43 (100)
*x* ^*2*^	6.362					
*P*	0.174					

**Table 2:** APACHE II score at admission, albumin level at admission, and prealbumin level at admission.

Group	*n*	APACHE II score at admission (±s)	Albumin level at admission (±s, g/L)	Prealbumin level at admission (±s, g/L)
Intervention group	44	22.7 ± 9.5	35.33 ± 6.58	0.175 ± 0.075
Control group	43	22.2 ± 6.1	36.19 ± 6.80	0.153 ± 0.069
*χ* ^*2*^ */t*		0.276	0.605	1.455
*P*		0.783	0.547	0.149

**Table 3:** Tests of within-subject effects.

Source		Type III sum of squares	d*f*	Mean squares	*F*	Sig
Treatment	Greenhouse-Geisser	243.929	1.000	243.929	5.107	0.039
Error (treatment)	Greenhouse-Geisser	716.460	15.000	47.764		
time	Greenhouse-Geisser	7.965	1.429	5.572	0.130	0.809
Error (time)	Greenhouse-Geisser	918.557	21.441	42.841		
treatment^*∗*^time	Greenhouse-Geisser	184.693	1.524	121.216	3.033	0.080
Error (treatment^*∗*^time)	Greenhouse-Geisser	913.303	22.855	39.961		

**Table 4:** GEE model parameter estimates of prealbumin.

Parameter	*B*	Standard error	95% CI	Wald *χ*^2^	d*f*	*P*
Intercept	0.187	0.012	0.164 to 0.210	254.789	1	0.000
Group	0.048	0.016	0.078 to 0.017	9.372	1	0.002
Measure time	0.002	0.001	−0.001 to 0.004	2.093	1	0.148
Group ^*∗*^measure time	0.001	0.002	−0.002 to 0.004	0.278	1	0.598

**Table 5:** Length of ICU stay and ventilator time (*n*, *M* (P25, P75), unit: day).

Group	Length of ICU stay	Ventilator time
Intervention	44, 6.4 (3.3, 10.7)	31, 3.6 (1.2, 6.0)
Control group	43, 7.9 (5.6, 12.1)	30, 5.5 (4.1, 8.8)
*Z*	−2.238	−2.179
*P*	0.025	0.029

**Table 6:** Complications (*n* (%)).

Group	Diarrhea	Gastric retention	Pulmonary infection
Control group (*n* = 43)	5 (11.6)	5 (11.6)	7 (16.3)
Intervention group (*n* = 44)	2 (4.5)	0 (0.0)	1 (2.3)
*x* ^*2*^	0.266	0.026	0.030
*P*			

## Data Availability

The datasets used or analyzed during the current study are available from the corresponding author upon reasonable request.
